# Geometrical confinement controls the asymmetric patterning of brachyury in cultures of pluripotent cells

**DOI:** 10.1242/dev.166025

**Published:** 2018-09-21

**Authors:** Guillaume Blin, Darren Wisniewski, Catherine Picart, Manuel Thery, Michel Puceat, Sally Lowell

**Affiliations:** 1 MRC Centre for Regenerative Medicine, Institute for Stem Cell Research, School of Biological Sciences, University of Edinburgh, Edinburgh, EH16 4UU, UK; 2 Univ. Grenoble-Alpes, CEA, CNRS, INRA, Biosciences and Biotechnology Institute of Grenoble, Laboratoire de Physiologie Cellulaire and Végétale, UMR5168, CytoMorpho Lab, 38054 Grenoble, France; 3 Univ. Paris Diderot, CEA, INSERM, Hôpital Saint Louis, Institut Universitaire d’Hematologie, UMRS1160, CytoMorpho Lab, 75010 Paris, France; 4 INSERM U1251, Université Aix-Marseille, MMG, 13885 Marseille, France

**Keywords:** Embryonic, Heterogeneity, Micropatterning, Self-organisation, Stem cells, Mouse

## Abstract

Diffusible signals are known to orchestrate patterning during embryogenesis, yet diffusion is sensitive to noise. The fact that embryogenesis is remarkably robust suggests that additional layers of regulation reinforce patterning. Here, we demonstrate that geometrical confinement orchestrates the spatial organisation of initially randomly positioned subpopulations of spontaneously differentiating mouse embryonic stem cells. We use micropatterning in combination with pharmacological manipulations and quantitative imaging to dissociate the multiple effects of geometry. We show that the positioning of a pre-streak-like population marked by brachyury (T) is decoupled from the size of its population, and that breaking radial symmetry of patterns imposes polarised patterning. We provide evidence for a model in which the overall level of diffusible signals together with the history of the cell culture define the number of T^+^ cells, whereas geometrical constraints guide patterning in a multi-step process involving a differential response of the cells to multicellular spatial organisation. Our work provides a framework for investigating robustness of patterning and provides insights into how to guide symmetry-breaking events in aggregates of pluripotent cells.

## Abstract (precis)

**Highlighted Article:** Asymmetric geometrical confinement guides polarised patterning and ensures positional precision of a primitive streak-like population of cells in mouse pluripotent cultures.

## INTRODUCTION

**Fig. 1.**
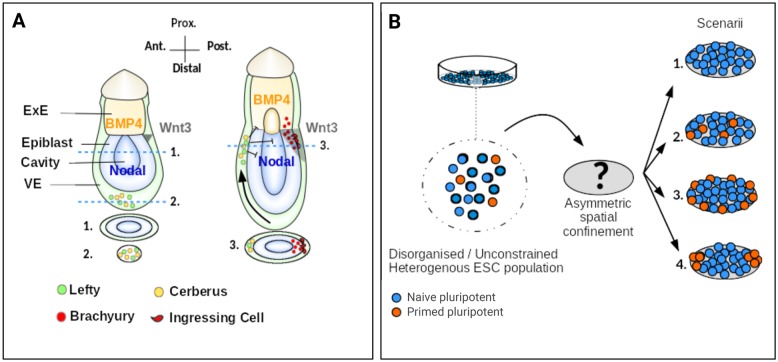
**Methodological approach and tested hypotheses.** (A) Schematic illustrating the emergence of AP polarity in the post-implantation mouse embryo. A sagittal section is drawn for each stage (top) as well as transverse sections (bottom) with numbered dashed lines indicating the positions of the represented sections. Note the ellipsoidal shape of the transverse sections. The black arrow represents the movement of the DVE cells towards the anterior side. (B) ESCs contain subpopulations with distinct expression profiles. Spatial confinement may (1) modify the balance of cell states, (2) have no apparent effect, (3) enable patterning via border effects in a symmetry-insensitive fashion or (4) enable patterning with geometry guiding spatial organisation. ExE, extra-embryonic ectoderm; VE, visceral endoderm.

Developmental patterning is the process through which spatially defined regions of distinct cell types emerge from a group of cells that initially appear to be equivalent. During early embryonic development, such a process requires a symmetry-breaking event in order to generate the first landmarks that will define the future axes of the body. In the mouse, antero-posterior (AP) polarity becomes apparent by early post-implantation stages (Fig. 1A). Wnt3 expression emerges in the proximo-posterior side of the embryo (Rivera-Pérez and Magnuson, 2005) and engages in a signalling autoregulatory loop involving Nodal from the epiblast and BMP4 from the extra-embryonic ectoderm (ExE) (Ben-Haim et al., 2006; Brennan et al., 2001). Nodal and BMP4 participate in the specialisation of distal visceral endoderm (DVE) cells (Kimura-Yoshida et al., 2005; Rodriguez et al., 2005; Yamamoto et al., 2004), which subsequently migrate towards the anterior side (Ding et al., 1998; Rodriguez et al., 2005; Srinivas et al., 2004) (reviewed by Stower and Srinivas, 2014). DVE cells are a source of cerberus, Lefty1 or Dkk1, which act as antagonists of the Nodal, BMP and Wnt pathways, and thus participate in a negative-feedback loop that restricts the activity of Nodal/Wnt/BMP to the posterior side of the embryo (Belo et al., 1997; Glinka et al., 1998; Kimura-Yoshida et al., 2005; Meno et al., 1996; Yamamoto et al., 2004). Gastrulation is apparent by embryonic day (E) 6.5 with the formation of the primitive streak (PS) under the influence of Wnt3 (Barrow et al., 2007; Liu et al., 1999; Yoon et al., 2015). The PS is characterised by the expression of early mesendodermal markers such as brachyury (T) (Beddington et al., 1992; Wilkinson et al., 1990; loss of epithelial characteristics reviewed by Morali et al., 2013) and an inversion of polarity prior to migration of ingressing cells (Burute et al., 2017; Stern, 1982).

Patterning was long thought to be restricted to *in vivo* development given the apparent disorganisation of differentiating pluripotent cells in culture. However, patterning events reminiscent of those in the embryo have been reported to occur within 3D aggregates of pluripotent cells (Brink et al., 2014; Harrison et al., 2017; Marikawa et al., 2009; ten Berge et al., 2008), indicating that it might be possible to recapitulate *in vitro* the self-organising competence of these cells. These remarkable findings call to mind the idea that early embryonic patterning may be formulated in engineering terms (Davies, 2017; Laurent et al., 2017; Sasai, 2013). Indeed, an interesting approach is to consider what would be the minimal set of external instructions required to allow pluripotent stem cells to recapitulate a normal developmental patterning programme. Pioneering studies with embryonic stem cells (ESCs) (Bauwens et al., 2008; Davey and Zandstra, 2006; Peerani et al., 2007, 2009) and with multipotent cells (McBeath et al., 2004) have shown that spatial confinement of colonies of cells on 2D patterns make it possible to harness and challenge the environment-sensing abilities of cells in culture. These studies have demonstrated the ability of stem cells to form their own niche, i.e. to generate their own gradients of morphogens and their competence to interpret signals in a position-dependent manner.

These founding works paved the way to the recent establishment of a method of recapitulating several aspects of the early gastrulating embryo in cultures of pluripotent cells (Etoc et al., 2016; Morgani et al., 2018; Tewary et al., 2017; Warmflash et al., 2014). These studies have started to identify the constraints on cell signalling and cell number required to generate patterns within *in vitro* cultures, thereby providing novel insights into the underlying mechanisms. However, patterns observed to date have been radially symmetric and leave open the question of whether the axis of an autonomous self-patterning event is sensitive to geometrical constraints and thus may be guided with engineered extrinsic cues. In the present work, we investigate geometrical confinement as a means of breaking radial symmetry (Fig. 1B).

We report that, indeed, the positioning of a pre-streak population marked by brachyury (T) depends on the geometry of the group of cells and that radial asymmetries in micropatterns result in radial asymmetric patterning of these cells. We adopted a multiscale and quantitative approach to reveal that positioning of T^+^ cells upon confinement is decoupled from the number of cells expressing T. We show that this number is defined by Wnt and Nodal signalling, similar to the mechanisms that establish AP polarity during embryonic development. We highlight the importance of culture history on the size of the T^+^ population and show that although the overall number of T^+^ cells is predictable at the level of the entire population, the proportion of T^+^ cells is highly variable within individual colonies. We demonstrate that geometrical confinement enables compound effects to guide patterning despite variable initial conditions. Finally, we discuss the implications of these findings for pattern formation in ESC aggregates and during gastrulation.

## RESULTS

### Geometry dictates T patterning in ESC colonies

The signals that control cell identity at gastrulation are well understood (Fig. 1A) but links between morphogenesis and differentiation are still unclear. Previous studies have shown that ESC cultures normally contain a population of cells expressing T protein (Suzuki et al., 2006), a transcription factor that emerges asymmetrically and marks the onset of gastrulation in embryos (Beddington et al., 1992; Wilkinson et al., 1990). However, during conventional 2D cell culture, no apparent spatial organisation is observed. *In vivo*, the morphology of the embryo likely provides spatial constraints to shape morphogen gradients and to guide morphogenetic processes. We hypothesised that the apparent spatial randomness observed in the dish is a consequence of the lack of geometrical confinement (Fig. 1B).

**Fig. 2.**
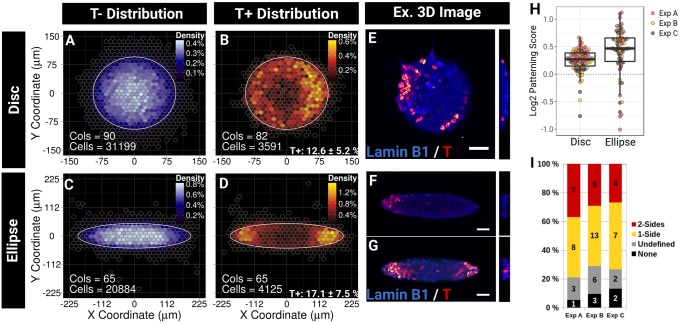
**Geometrical confinement guides the positioning of T^+^ cells.** (A-D) BDMs of the T^−^ or T^+^ populations. Cells, total number of cells; Cols, total number of colonies. (E-G) Representative confocal images of ESCs grown on disc (E) or ellipse (F,G) micropatterns with a *yz* section on the right. A one-sided (F) and a two-sided (G) colony are shown. Scale bars: 50 µm. (H) Variability of the patterning score across individual colonies. Data points are colour-coded by experimental replicates. The box plot indicates the median and the intra-quartile range (IQR). Whiskers indicate the inner fence (1.5 IQR). (I) Proportion and number of colonies grown on ellipse micropatterns falling into each patterning categories. Undefined, random positioning of T^+^ cells; None, no T^+^ cells found in the colony. All results are shown for three independent experiments. Ex., example.

To test this idea, we developed a method to determine the preferential distribution of T^+^ cells in colonies (Fig. S1). We used micropatterns to provide geometrical constraints by allowing us to control precisely the shape and size of the area on which cells can adhere and grow. We took the approach of analysing multiple colonies in order to map the average preferential localisation of the cells within each shape in the form of a binned density map (BDM). When cells were grown on 30,000 µm^2^ circular discs or ellipses for 48 h with a 400 µm pitch between patterns (Fig. 2 and Fig. S2), ESCs fully colonised the pattern and formed dome-shaped colonies, resulting in a radial gradient of cell densities with highest cell densities in the centre of the shape. This effect is shown by the BDM of T^−^ cells, which represented the majority of the population (we found on average 12.6±5.2% of T^+^ cells per disc and 17.1±7.5% of T^+^ cells per ellipse) (Fig. 2A-D and Fig. S3) and by the *z* projection of 3D confocal images (Fig. 2E-G). Strikingly, on disc micropatterns, the BDM of T^+^ cells revealed that T^+^ cells were preferentially located at the periphery of the group at an average distance of 34.8 µm from the boundary of the shape (62.7 µm from the centre) (Fig. 2B,E). Remarkably, on ellipse micropatterns, T^+^ cells did not localise on the entire circumference of the shape but instead were positioned at the tips only, at an average distance of 11 µm from the tip (109 µm from the centre; Fig. 2D,F,G).

In order to quantify the reproducibility of T^+^ cells positioning across individual colonies, we computed a ‘patterning score’ for each colony (see Materials and Methods). The distribution of the patterning score confirms that patterning occurs in the majority of the shapes (85%) both for discs and ellipses with only 15% of the T^+^ colonies having T^+^ cells randomly positioned or closer to the centre than average (Fig. 2H,I and Fig. S3). These distributions also show that the difference between the positioning of T^+^ cells compared with average positioning of every cell is more pronounced on ellipse patterns indicating that the radial asymmetry introduced with the ellipse reinforces the pattern. There was, however, less inter-colony variability on discs than on ellipses, which may be explained by the higher range of possible distances from the centre on ellipses. Strikingly, T^+^ cells did not always distribute on both sides of the ellipse: T^+^ cells were positioned on the two sides in 35% of the cases and on only one side in 40% of the cases (Fig. 2F,I, Fig. S2).

Taken together, our results demonstrate that patterning of T^+^ cells may be guided in part by geometrical confinement and that patterning is not explained by border effects alone as elliptical shapes contribute to breaking of the radial symmetry of patterning.

### Global cell density dominates over local interactions to predict the percentage of T^+^ cells

Cell density may influence both chemical and physical aspects of the microenvironment. Therefore, to obtain further insights into the mechanisms underlying patterning in culture, we decided to first investigate whether cell density could influence T expression and, if so, at which length scale.

**Fig. 3.**
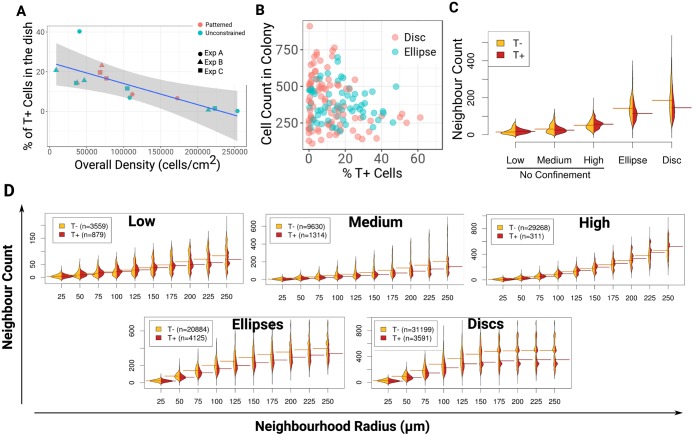
**Bulk cell density defines the percentage of T^+^ cells** **and** **cell clustering imparts T patterning.** (A) Evolution of the percentage of T^+^ cells with global cell density. A linear regression fitted on unconfined cultures data points is shown with the shaded region indicating the 95% confidence interval. (B) Percentage of T^+^ cells versus the total number of cells within each colony grown on disc or ellipse micropatterns. (C,D) Split bean plots of the distributions of the neighbour count around each T^−^ (orange) or T^+^ (red) cells. Horizontal lines indicate the median. (C) Experimental conditions comparison using a fixed radius (75 µm). (D) Comparison of increasing radii for each condition.

We plated cells at low (2000 cells/cm^2^), medium (10,000 cells/cm^2^) or high (50,000 cells/cm^2^) density within standard culture dishes in order to create varying distributions of local densities in the dish. Because this would also change the total number of cells per volume of medium (global density) we also cultured the cells on disc M and ellipse M micropatterns (Fig. S2) to enforce cell clustering for a global density expected to be equivalent to the medium density of unpatterned cultures. This served to decouple effects of global density from the effects of local densities (Fig. 3 and Fig. S4).

We first measured the percentage of T^+^ cells by quantitative immunofluorescence (qIF) and plotted this against surface coverage. The negative correlation shown in Fig. 3A demonstrates that lower global density results in a higher percentage of T^+^ cells. Surprisingly, culturing the cells on patterns in order to increase local density without increasing global density did not noticeably affect the overall percentage of T^+^ cells in response to the changes in global density. Also, when we plotted the total number of cells against the percentage of T^+^ cells per pattern for both discs and ellipses (Fig. 3B), we observed a large inter-pattern variability and did not find any convincing correlation, indicating that standardising the size, shape and distance between colonies was not sufficient to normalise the percentage of T^+^ cells per colony.

We next constructed the distribution of local cell densities for each condition by computing the number of neighbours found within a circular region around each cell. To compare all conditions together, we first fixed the neighbourhood radius (NR) to 75 µm (Fig. 3C, Fig. S4), a distance slightly smaller than the radius of the disc micropatterns. As expected, the average local densities found on patterns largely exceeded the highest local densities found within unconfined cultures, confirming that micropatterns enforced cell clustering (Fig. 3C) while maintaining a global density similar to the medium unconstrained culture (Fig. 3A). Although confinement did not influence the overall percentage of T^+^ cells, it imparted the preferential localisation of T^+^ cells to the regions of lowest local density (Fig. 3B), an effect that was not apparent with unconstrained cultures. To understand this result better, we tested NRs ranging from 25 to 250 µm (Fig. 3D). The positioning of T^+^ cells to lower densities became increasingly apparent as we increased NR. Interestingly, the NR value required to observe an effect decreased with the amount of clustering in the culture (200 µm for low, 125 µm for medium and 50 µm for patterns) and matched with a relatively similar range of neighbours count in each condition (0 to ∼200 cells, Fig. 3D).

Taken together, our results demonstrate that global cell density influences the number of T^+^ cells in the culture but not their pattern. Patterning does, on the other hand, correlate with local variations in cell density that can be enforced by confinement.

### A latency effect contributes to local variability in cell fate

We next set out to understand the global effects that influence T expression. Notably, we observed considerable variability in the numbers of T^+^ cells per pattern despite the reproducible linear correlation observed when combining data from multiple patterns (Fig. 3B). We wondered whether this variability may be explained by a latency effect, i.e. by the fact T expression depends on past as well as present global density.

**Fig. 4.**
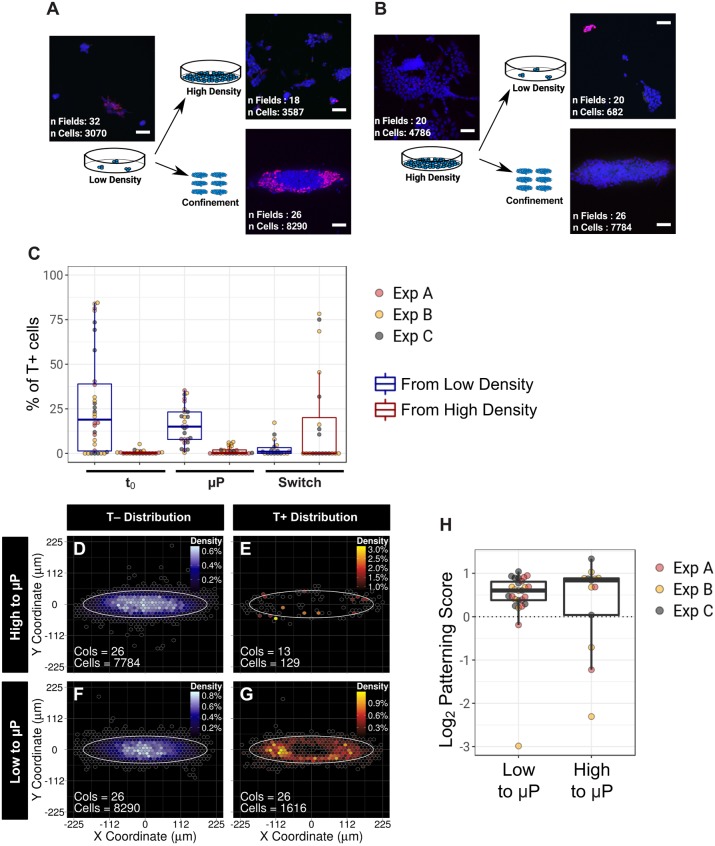
**The size of the T^+^ population is a consequence of both current and past culture density.** (A,B) Overview of the ‘memory test’ experiment. Sample images for each condition are shown (red, Tbra; blue, DAPI). n Fields, number of fields taken into account; n Cells, number of detected cells across three independent experiments. (C) Percentage of T^+^ cells observed in the ‘memory test’ experiment. Each data point corresponds to the percentage of T^+^ cells observed in one field of view. The colours of the box plots indicate whether the cells have been pre-cultured at low or high density. t0, observation before re-plating the cells; µP, 48 h on micropatterns; switch, 48 h at the opposite density (unconstrained). (D-G) BDM of T^−^ and T^+^ cells grown on ellipse micropatterns after pre-conditioning the cells at either low or high density. (H) Variability of the patterning score observed for individual ellipses. In C and H, data points are colour-coded by experimental replicates.

To test this, we designed a ‘memory test’ by pre-culturing the cells at either low or high density for 48 h before plating them at the opposite density or on ellipse micropatterns for an additional 48 h (Fig. 4A,B).

We found that the proportion of T^+^ cells in the culture was only partially reversible when switching to the opposite extreme density (Fig. 4C, ‘switch’). Indeed, when switching from low to high density, the overall percentage of T^+^ cells remained low (as indicated by the median in Fig. 4C) with only a minor fraction of the colonies containing a percentage of T^+^ cells similar to colonies present at low density at t0. This indicates that culture conditions experienced by the cells in the previous passage have an effect on the number of T^+^ cells for more than 48 h. Strikingly, when the cells were plated on micropatterns, this memory effect was the main predictor of the percentage of T^+^ cells with only a minor influence of final density after re-plating (Fig. 4C). Importantly, T patterning was not affected by the initial percentage of T^+^ cells (Fig. 4D-H).

To understand better how global density may shift the relative proportion of each cell population, we monitored the expression of early developmental genes (Fig. S5A,B). Several pro-differentiation factors mark subpopulations of ESCs with biases towards specific routes of differentiation (Canham et al., 2010; Davies et al., 2013; Niakan et al., 2013; Singh et al., 2007). We thus decided to investigate the regulation of genes that identify embryonic domains at the onset of gastrulation. We found that the levels of the proximal markers *T* and *Wnt3* (Rivera-Pérez and Magnuson, 2005) were negatively correlated with cell density whereas the AVE markers cerberus 1 (Belo et al., 1997) and *Foxa2* (Kimura-Yoshida et al., 2007) were positively correlated with cell density. These results suggest that lower densities favour proximo-posterior identity in ESCs, whereas high density favours an environment permissive for anterior lineages.

We next investigated whether the effect described above could be attributed to diffusible signals. We found that conditioned media (CM) from cells grown at high density had a dose-dependent effect on the expression of *T*, *Wnt3* and cerberus 1 in cells cultured at low density, indicating that diffusible signalling molecules contribute to regulation of these genes. Conversely, *Lefty1* and *Foxa2* expression was not affected by the addition of CM, opening the possibility that local mechanical cues or juxtacrine signalling could be required to modulate their expression (Fig. S5C).

In agreement with a previous report (Kempf et al., 2016), our results support the idea that ESCs secrete molecules that can inhibit posterior fates. Increasing the overall cell density may increase inhibitor concentration and shift the preferential cell identity in the population. This shift can then have consequences on the cells' response during the subsequent passage. Furthermore, this ‘latency effect’, which may relate to lineage priming (Tsakiridis et al., 2014), might explain the inter-pattern variability that we observed (Fig. 3B). Indeed, owing to the small number of cells that each pattern receives upon plating, the initial proportions of T^+^ cells per pattern may vary significantly. Because of the latency in cell fate change, this initial difference could explain the variability observed at the end of the experiment.

### Reduced distance between colonies does not alter the percentage of T^+^ cells or their positioning

**Fig. 5.**
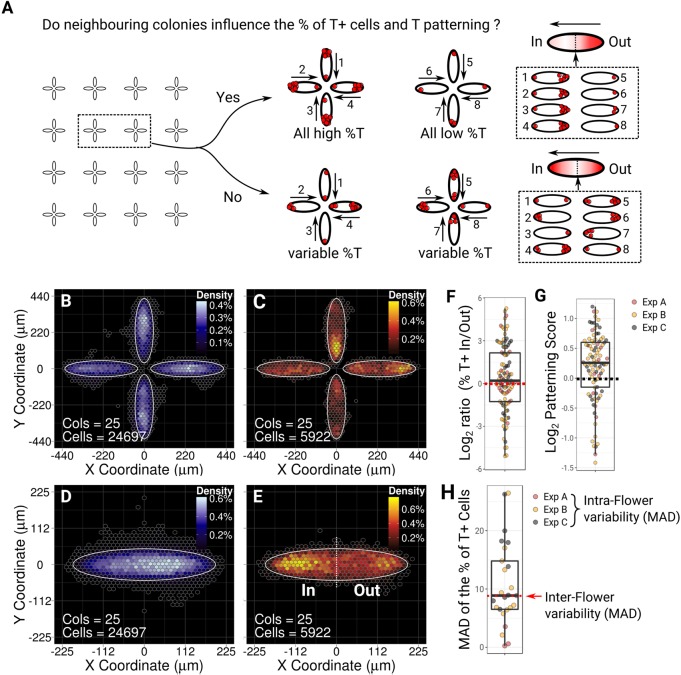
**The size of the T^+^ population is a consequence of both current and past culture density.** (A) Possible outcomes of the flower experiment. In each case, two flowers are shown to illustrate how the percentage and patterning of T^+^ cells should vary. On the right-hand side, the expected BDM obtained from the registration of all individual ‘petals’ is shown. The numbers in the diagram on the right match the petal number in the example flowers in the middle of the schematic with the arrow pointing towards the ‘inner’ tip of the ellipse. (B-E) BDMs for T^−^ (B,D) and T^+^ cells (C,E). (F) Distribution of the inner/outer tips ratio of the percentage of T^+^ cells (log_2_). (G) Variability of the patterning score across flower petals. (H) Distribution of the mean absolute deviation from the mean (MAD) for the percentage of T^+^ cells found within petals of each flower. The red dashed line and arrow indicate the value of the MAD found across flowers. All results include three independent experiments (colour-coded by experimental replicates).

The fact that secreted molecules inhibit T expression opens the possibility that the restriction of T^+^ cells to the low-density regions is a consequence of lower inhibitor concentration. In order to test this hypothesis, we designed a shape that consisted of four ellipses arranged as a four-petalled flower with a separation distance of 600 µm between flowers (Fig. 5A and Fig. S2). We reasoned that if all the cells secrete inhibitors at a constant rate (Nelson et al., 2006), inhibitors would become more concentrated at the centre of the flower where colonies are the closest to one another rather than on the periphery.

To maximise the effect of our design, we used the minimal distance between petals that prevented the colonies from merging together during the course of the experiment (25 µm, Fig. S2). We used neighbourhood maps as a proxy for the putative concentration profiles of inhibitors. Fig. S6 shows that a higher concentration of inhibitors at the centre may be expected if signals are diffusing over a distance of ∼100 µm or above. If this prediction was correct, one would expect a lower number of T^+^ cells at the centre of the flower than at the periphery. However, we did not observe such an effect (Fig. 5B-F). Instead, the relative proportion of T^+^ cells found on the inner tips rather than the outer tips of the ellipses was variable with the median slightly above 0, indicating that, if anything, inner tips were slightly enriched in T^+^ cells (Fig. 5F). Also, patterning in this configuration was not affected (Fig. 5G). Finally, if inhibitors diffused over a distance similar to the flower size, a lower variability in the percentage of T^+^ cells amongst the petals of the same flower compared with the variability across flowers may be expected (Fig. 5A). Again, we observed a diversity of variabilities within each flower pattern (Fig. 5H) with the median of the distribution of mean absolute differences (MAD) being equal to the mean of the MAD found across flowers.

Taken together, these results indicate that neighbouring colonies do not influence each other across the distances tested. Inhibitors may diffuse only over a short range (<100 µm) or alternatively may diffuse over a much longer range to become near-homogenous across the dish.

### Nodal, Wnt and Fgf signalling regulate the emergence and positioning of T^+^ cells in culture

**Fig. 6.**
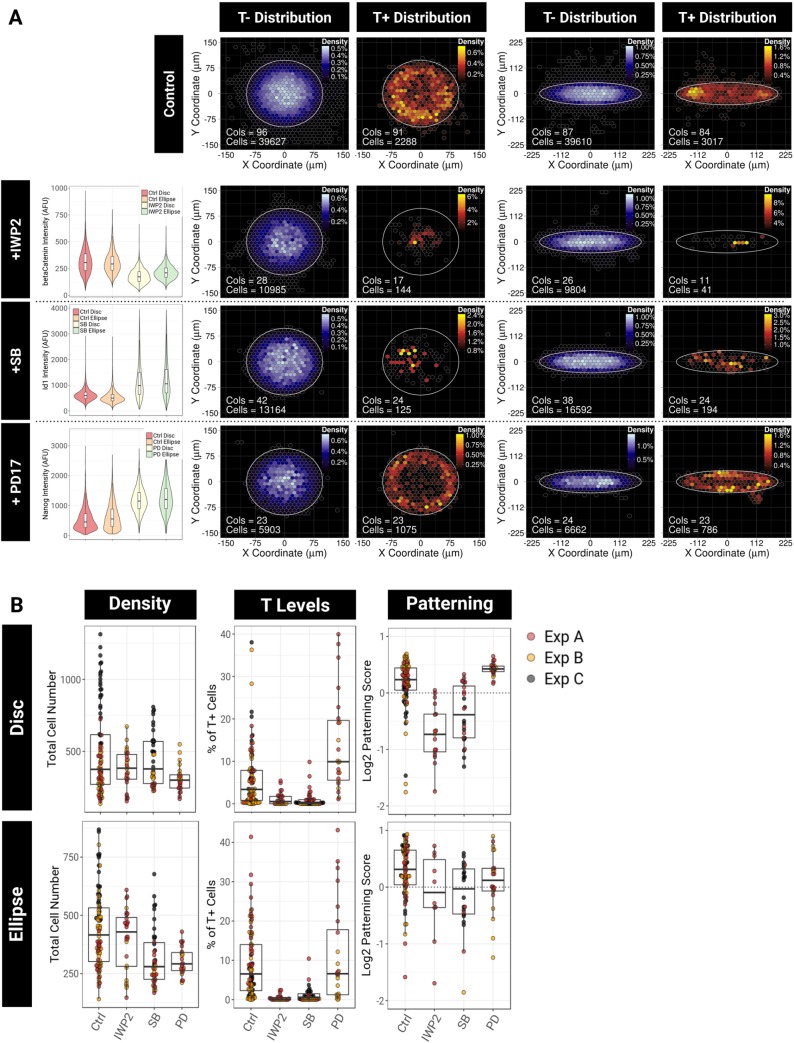
**Nodal, Wnt and FGF signalling regulate the emergence and positioning of T^+^ cells in the culture.** (A) BDMs of the localisation of T^−^ and T^+^ cells for colonies grown on disc or ellipse micropatterns with or without 48 h pathway inhibitor treatment. SB, 10 µM SB-431542 (Nodal/activin inhibitor); IWP2, 5 µM (Wnt inhibitor); PD17, 100 nM PD-173074 (Fgf inhibitor). Violin plots of the distribution of qIF intensities of a pathway reporter are shown on the left of BDMs. AFU, arbitrary fluorescence unit. (B) Beeswarm box plots representing, for discs (top row) and ellipses (bottom row), the total cell number per colony, the percentage of T^+^ cells and the log_2_ of the patterning score. Colours indicate three independent experiments.

In order to determine which pathways regulate the number of T^+^ cells, we cultured cells on disc and ellipse micropatterns in the presence of inhibitors known to alter AP polarity *in vivo* (Fig. 6). Inhibitors were added at the time of seeding cells onto micropatterns. For each pathway, we measured the expression of a downstream readout using qIF (violin plots in Fig. 6A). This allowed us to confirm the expected inhibition and to assess the endogenous activity of the pathways that we tested.

We first disrupted the canonical Wnt pathway using IWP2, which inhibits secretion of Wnt ligands (Chen et al., 2009). We observed a decrease in the level of nuclear β-catenin (Fig. 6A, second row), indicating that the inhibitor is functional and that canonical Wnt signalling is otherwise active in these conditions. Wnt inhibition resulted in a strong reduction in the number of T^+^ cells (Fig. 6B) and a preferential localisation of the remaining cells close to the centre of the colony (Fig. 6A,B).

Next, we inhibited Nodal/activin signalling using the small molecule SB431542 (SB). Id1 is negatively regulated by Nodal (Galvin et al., 2010), so we used Id1 as an inverse readout of Nodal activity. We found a strong increase in the level of Id1 upon treatment with SB (Fig. 6A, third row) suggesting that Nodal signalling is strongly active under basal culture conditions, in agreement with previous reports (Ogawa et al., 2007; Papanayotou et al., 2014). Similarly to Wnt inhibition, disruption of Nodal/activin signalling resulted in a severe drop in the number of T^+^ cells (Fig. 6B) as well as a randomisation of their localisation (Fig. 6A,B). Our results demonstrate that both Wnt and Nodal signalling are required for T expression and for T patterning.

BMP inhibition had no influence on either Id1, which is a direct target of the pathway (Hollnagel et al., 1999), or T expression (Fig. S7) indicating a lack of autocrine BMP: this pathway was therefore excluded from further analysis. This observation is in line with previous reports showing that BMP activity is not required to activate T once Nodal and Wnt signalling are active (ten Berge et al., 2008; Turner et al., 2014b).

Finally, we inhibited Fgf using 100 nM of PD173074 (PD17), an Fgf receptor tyrosine kinase inhibitor. Nanog is negatively regulated by autocrine Fgf signalling (Yamanaka et al., 2010) and so serves as an inverse readout of Fgf activity. As expected, we found a strong increase in Nanog expression upon PD17 treatment, consistent with the fact that Fgf4 is abundantly secreted by ESCs (Kunath et al., 2007). Fgf inhibition reinforced patterning on disc micropatterns (Fig. 6A): first it reduced the proportion of T^+^ cells in the middle of the shape and second it strongly reduced the variability in the positioning of T^+^ cells across individual colonies (Fig. 6B). Notably on ellipse micropatterns, T^+^ cells were excluded from the middle of the shape but failed to localise at the tips, and instead, remained positioned around the border of the shape (Fig. 6A, bottom-right panel).

Altogether, these results demonstrate that the mechanisms regulating T expression in this system resemble those that position the primitive streak in the embryo, and suggest that the positioning of T on micropatterns requires the concerted action of autocrine Nodal, Wnt and Fgf signalling whereas BMP activity appears to be dispensable.

These findings also show that T patterning is generated by at least two processes that can be decoupled: Wnt and Nodal signalling are required to maintain the pool of  T^+^ cells in the culture and to position the cells in a local density-sensitive manner, and Fgf signalling is required for the restriction of the T^+^ cells to the tips of the ellipses.

### T patterning does not emerge until after cell confluency and does not require EMT

Wnt activity and T expression precede an epithelial-to-mesenchymal transition (EMT) *in vivo* (Carver et al., 2001; Williams et al., 2012) and *in vitro* (Turner et al., 2014a). We therefore hypothesised that EMT may be involved in the positioning of T^+^ cells. To test this idea, we quantified the number of T^+^ cells co-expressing pluripotency- or EMT-associated markers (Fig. S8). Nanog and Oct4 (Pou5f1) are pluripotency-associated factors that are downregulated as cells ingress into the streak (Osorno et al., 2012). We found that the vast majority of T^+^ cells co-expressed Oct4 (Fig. S8A,B) and that 75% of T^+^ cells were also expressing Nanog (Fig. S8C,D). T^+^ cells did not express Snail1 (Snai1), which drives EMT at the streak (Acloque et al., 2011; Cano et al., 2000; Carver et al., 2001). Finally, we observed that only a small fraction of T^+^ cells expressed N-cadherin (cadherin 2) (Fig. S8G), an early marker of EMT (Radice et al., 1997). Altogether, these observations indicate that T^+^ cells in the culture are still at an early stage of differentiation and have not yet undergone full EMT.

**Fig. 7.**
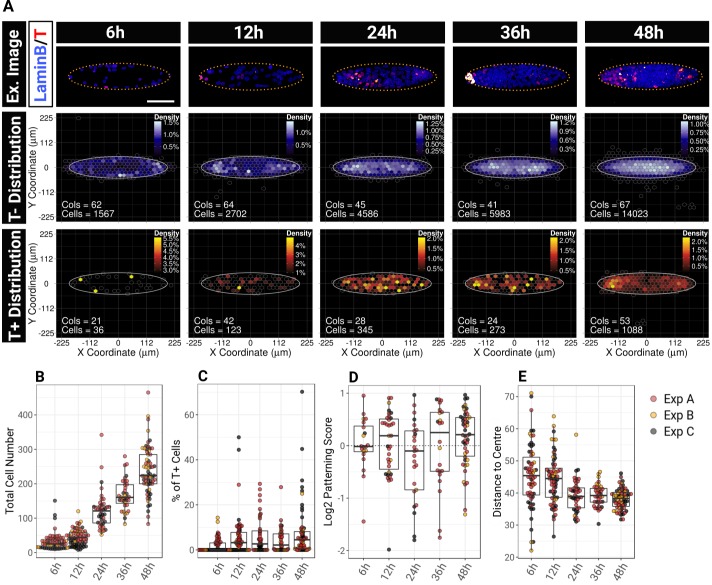
**T patterning becomes apparent 36 h after plating when cell density becomes more important in the middle of the shape.** (A) Sample confocal images and BDMs of the localisation of T^−^ and T^+^ cells observed 6 h, 12 h, 24 h, 36 h and 48 h after plating. The dotted yellow line on the sample images indicate the contour of the pattern. Scale bar: 100 µm. (B-E) Beeswarm box plots representing the evolution of the total number of cells (B), the percentage of T^+^ cells (C), the patterning score (D) and the average distance of all the cells from the centre (E) for individual colonies at each time point across three independent experiments (colour-coded by experimental replicates).

We next monitored how patterning becomes established over time after plating ESCs on ellipse micropatterns (Fig. 7). Cells become confluent around 24 h after plating (Fig. 7A) with an average of 110 cells per colony (Fig. 7B). Consistent with our ‘memory test’ experiment, the percentage of T^+^ cells does not vary significantly over time despite some variability within individual colonies. Importantly, we found that T patterning became apparent from 36 h (Fig. 7D). Therefore, the segregation of T^+^ cells to the tips of the ellipse occurs at least 12 h after confluency and is progressively reinforced as cell density builds up in the middle of the shape (Fig. 7A,E).

Together, these results indicate that T^+^ cell positioning is a dynamic process during which the proportion of T^+^ cells remains constant and the increase of local cell density precedes the restriction of T^+^ cells to the tips of the ellipse.

### Shape, scale and geometry dictate asymmetries in the local densities, which in turn guide T^+^ cell positioning

Does the strong curvature at the tips of the ellipse guide T^+^ cell positioning? Numerical simulations have suggested that cells experience high tension where micropattern convex curvature is highest (Nelson et al., 2005) and both tension and curvature may regulate cell fate (Dupont et al., 2011; Engler et al., 2006; McBeath et al., 2004; Ruiz and Chen, 2008; Varelas et al., 2010) and guide directional movements (Rausch et al., 2013; Ravasio et al., 2015; Rolli et al., 2012).

**Fig. 8.**
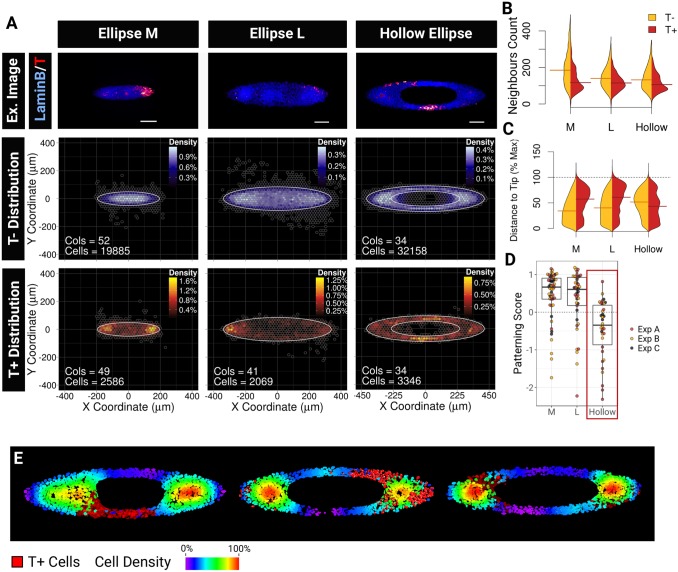
**Shape, scale and geometry** **dictate asymmetries in the local densities which in turn guide T^+^ cells positioning.** (A) Confocal images examples (Ex. Image) and BDMs for T^−^ and T^+^ cells grown on ellipse M, ellipse L and hollow ellipse micropatterns. Scale bars: 100 µm. (B) Comparison of the distributions of neighbours counts within a 75 µm radius (B) or of the relative distance from the tip of the ellipse (C) for T^−^ (orange) and T^+^ (red) cells and for each type of ellipse tested. Horizontal bars indicate the median. (D) Distributions of the log_2_ of the patterning score across colonies for each pattern shape. M, ellipse M; L, ellipse L; hollow: hollow ellipse. Red box highlights the patterning inversion on hollow ellipses. (E) Sample heat maps of the cell density (75 µm radius) found on hollow ellipses. Note the positioning of T^+^ cells (bright red), which can be found on a single branch of the ellipse and which are always excluded from the high-density regions.

**Fig. 9.**
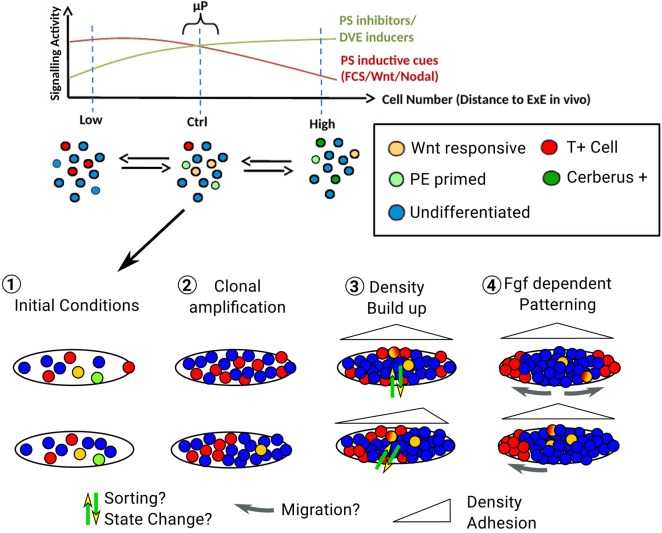
**Proposed speculative model.** The graph illustrates how global density modulates signalling activity and results in a balanced proportion of the various cell identities in the culture. In the absence of confinement, cells appear to be spatially disorganised. However, providing the cells with geometrical confinement reveals self-patterning of the T^+^ and T^−^ populations. On ellipsoidal micropatterns, two main outcomes in equal proportions can be observed: (1) segregation of T^+^ cells on both sides of the ellipse or (2) on one side only. We propose that cellular diversity together with seeding heterogeneity create small variations in the initial conditions of each micropattern within the same dish (1). Clonal amplification leads to the maintenance of the initial proportion of the T^+^ cells on the pattern, a mechanism that requires Wnt and Nodal activity. This phenomenon amplifies differences in the number of T^+^ cells across patterns (2). As the cells continue to proliferate, T^−^ cells start to form regions of high density from which T^+^ cells become excluded (3). Finally, an Fgf-dependent mechanism (possibly involving differential motile behaviours) leads to the segregation of T^+^ cells to the tips of the ellipses (4). PE, primitive endoderm.

Results from Fig. 7 suggest that the positioning of T^−^ cells towards the middle of the colony is important for T patterning. To determine whether it is density or curvature that predominantly regulates T^+^ cells patterning, we designed pattern shapes which would allow us to uncouple the effect of boundary curvature from the positioning of T^−^ cells (Fig. 8). We reasoned that a hollow ellipse shape would partition the T^−^ population into two high-density regions at the tips of the ellipse. We first tried to pattern cells using a hollow ellipse with the same dimensions as the plain ellipse M. Unfortunately, ESCs rapidly overgrew the pattern centre. We therefore decided to investigate whether increasing the size of the ellipse would lead to a mislocalisation of T^+^ cells. We found that the range of local densities experienced by cells was higher on small ellipses than on large ellipses (Fig. 8B), but did observe any difference in the patterning score (Fig. 8D). This result shows that T^+^ cell patterning scales with colony size. In contrast, when we plated cells on large hollow ellipses, the positioning of T^+^ and T^−^ cells was reversed with T^+^ cells localising towards the centre of the shape (Fig. 8C-E). This result argues against the curvature hypothesis and instead supports a model in which geometry guides the distribution of local cell densities, which in turn dictate the localisation of T^+^ cells, with T^+^ cells being excluded from high-density regions (Fig. 9).

## DISCUSSION

### Locally acting mechanisms may consolidate patterning events

Using micropatterns and quantitative imaging, we have shown that geometrical control of ESC colonies leads to the establishment of spatial patterns of T expression, including breaking radial symmetry.

These results are broadly in line with previous reports showing that self-organisation of differentiating cells can be observed with confinement (Etoc et al., 2016; Morgani et al., 2018; Tewary et al., 2017; Warmflash et al., 2014). However, the processes investigated here differ significantly. In previous studies (Etoc et al., 2016; Morgani et al., 2018; Warmflash et al., 2014), unspecified cells were released from their pluripotent states by directing their differentiation with morphogens. Cells were cultured on wide (1 mm diameter) discoidal micropatterns, which allowed for large domains (>100 µm) of gene expression to appear over time in response to emergent gradients of endogenous diffusible signals. This system has therefore been used to gain insights into the mechanisms of positional information and domain specification (Heemskerk et al., 2017 preprint; Tewary et al., 2017).

In the present study, we interrogate a distinct aspect of pattern formation. Phenomenon such as neighbour exchange in the epiblast (Ichikawa et al., 2013; Ramkumar et al., 2016) as well as non-uniform response threshold of individual cells to signals (Stevense et al., 2010) may lead to poorly defined domain boundaries (Lander, 2011). Noticeably, a ‘salt and pepper’ pattern of T expression is often apparent on the posterior side of the embryo, yet the positioning of the streak is precise at the cellular scale (Ramkumar et al., 2016; Williams et al., 2012). We therefore investigated whether positional precision requires additional mechanisms and whether such mechanisms may be leveraged to guide polarised patterning in culture.

### Small-scale geometrical confinement resolves patterning without altering the size of the T population

To test whether local tissue geometry refines patterning when spatially disorganised populations of cells are initially present, we used ESCs, which are inherently heterogeneous (Canham et al., 2010; Chambers et al., 2007; Davies et al., 2013; MacArthur and Lemischka, 2013; Toyooka et al., 2008) in combination with small asymmetric patterns (<90,000 µm^2^) and asked two questions: (1) can spatial standardisation normalise the proportion of a specific subpopulation and (2) can geometry guide spatial organisation of cells?

We found a strong variability in the number of T^+^ cells across colonies (Figs 3B, 6B and 7C). This variability was likely the result of initial differences in seeding densities and proportions of T^+^ cells (Fig. 7C) compounded by population memory effects (Fig. 4). Thus, although micropatterns standardise the size, shape and distance between colonies, micropatterns were not sufficient to regularise the percentage of T^+^ cells per colony. In contrast, radially asymmetric geometrical confinement did define radially asymmetric positioning of T^+^ cells, and did so independently of the initial proportion and distribution of T^+^ cells (Figs 2, 4 and 7). This decoupling of patterning from the size of the T population demonstrates that mechanisms regulating the positioning of T^+^ cells exist at a local level and raise the possibility that in the embryo, long-range patterning signals are modulated by local geometrical cues to correct for mis-specification events to ensure the robustness of the positioning of the primitive streak.

### Can diffusible signals fully explain the positioning of T^+^ cells?

We have demonstrated that the role of geometry in guiding radially asymmetric patterning of T^+^ cells is to control the distribution of local cell densities and that it does not act by directly triggering T expression as a result of mechanosensing at boundary curvature (Fig. 8). This raises the question of how local density regulates T positioning.

Our data indicate that ESCs secrete inhibitors of posterior fates (Fig. S5), in agreement with previous reports (Kempf et al., 2016). One possibility is that local cell density defines the local concentration of secreted inhibitors, which would in turn re-adjust T expression depending on the cell position in the colony.

To address this point, we needed to determine the distance at which inhibitors could influence T expression in comparision with the size of the micropatterns that we have used. Neighbour analysis (Fig. 3D) indicated that inhibitors diffuse over long distances: a correlation between T expression and density became apparent using a neighbourhood radius of at least 200 µm at low density. Furthermore, our flower experiment showed that elliptical colonies in close proximity did not influence each other's pattern or the number of T^+^ cells (Fig. 5). Finally, the absence of a clear correlation between the total number of cells per colony and the number of T^+^ cells (Fig. 3B) suggested that the mechanisms that define patterning are distinct and separable from the mechanisms that determine the percentage of T^+^ cells. Together, our data argue against a model in which a simple, colony-sized, emergent gradient of inhibitors restricts T-expressing cells to the tips of the ellipses.

We do not exclude the possibility that a more complex model of pattern formation; for example, a Turing mechanism (Briscoe and Small, 2015; Turing, 1952) may be compatible with our data. Further work will be necessary to determine this and we anticipate that numerical simulations together with a direct measurement of the dynamics of the system will help address the question.

### Does spatial reorganisation ensure positional precision?

An alternative mechanism that could explain the complex picture revealed by our multiscale approach is the spatial reorganisation of the T^+^ cells initially present at the start of the experiment.

Absence of Snail1 indicates that T^+^ cells have not yet undergone full EMT (Fig. S8); however, this does not preclude the possibility that cell-sorting events and differential motile behaviours occur precociously during the early steps of cell fate specification (Turner et al., 2014a). Indeed, the main drivers of T expression, Nodal and Wnt, can induce biophysical changes (Krieg et al., 2008; Reintsch et al., 2005; Trichas et al., 2011), which can be sufficient to drive cell sorting (Lecuit, 2008; Maître et al., 2016). It is therefore plausible that one of the roles of Nodal and Wnt in this system is to facilitate the exclusion of the T^+^ cells from high-density regions by cell sorting. This idea would fit with the observation that differential positioning of T^+^ cells is apparent at the streak before EMT (Burute et al., 2017; Ramkumar et al., 2016), as well as with the findings that neighbour exchange together with Nodal dependent community effects define which cells eventually ingress within the streak (Voiculescu et al., 2014).

Inhibition of Fgf had a clear effect on the positioning of T^+^ cells. It reinforced patterning on discs while impairing the restriction of T expression to the tips on ellipses (Fig. 6). Fgf promotes motile behaviours in multiple contexts (Ciruna and Rossant, 2001; Deng et al., 1994; Sun et al., 1999; Yamaguchi et al., 1994; Yang et al., 2002). Interestingly, it has been suggested that a gradient of a chemotaxic cue is not necessarily required to induce patterning and that a change in directional persistence could lead to cell sorting (Mori et al., 2009). It is therefore possible that Fgf signalling acts by triggering differential motile behaviours in this system as well.

Although further work will be required to determine whether spatial reorganisation is indeed the mechanism underlying T patterning on ellipses, this mechanism allows the formulation of a hypothetical model that is consistent with all our observations. This model is shown in Fig. 9. Inhibitory signals may reach a near-homogeneous concentration throughout the culture explaining why global density remains the best predictor of the overall percentage of T^+^ cells (Fig. 3). Culture history, i.e. past culture density, impacts the proportion of the multiple subpopulations present in the culture (Fig. 4) and dictates the non-uniform response threshold of the cells to global signals (see Chubb, 2017 for a discussion on mechanisms which could lead to this effect). Finally, as diffusible signals define the identity of the cells, the same signals also induce the acquisition of new biophysical properties to induce spatial reorganisation (Steinberg, 1963; Townes and Holtfreter, 1955) in a local density-sensitive manner. Such mechanism would also explain the stochastic occurrence of polarised patterns of T expression on ellipses as a result of a heterogeneous distribution of cell types on each side of the ellipse.

In this model, the regulation of T expression and T positioning constitute two coordinated but separate processes, thus removing the need for a tight coupling of patterning to the size of the population (Fig. 3B).

## Conclusion

In conclusion, we have quantified a novel effect of geometry in guiding the radially asymmetric patterning of otherwise disorganised cells. These findings raise the possibility that similar fine-tuning mechanisms help secure positional precision at the streak. Importantly, the decoupling of positioning and domain size suggests that multiple processes act in concert but at distinct scales. Our work provides a novel framework and experimental system to address this question in more detail. Our findings also raise the possibility of leveraging the principles described here in order to guide the polarised patterning of engineered stem cell assemblies (Laurent et al., 2017).

## MATERIALS AND METHODS

### Cell culture

CGR8 mouse embryonic stem cells were routinely maintained on gelatinised (Gelatin, Sigma) culture vessels (Corning) in Glasgow Minimum Essential Medium (GMEM, Sigma) supplemented with 10% foetal calf serum (FCS, APS), 100 U/ml LIF (produced in-house), 100 nM 2-mercaptoethanol (Gibco), 1× non-essential amino acids (Gibco), 2 mM L-glutamine (Gibco), 1 mM sodium pyruvate (Gibco). Cell culture was maintained at 37°C with 5% CO_2_ and routinely tested for mycoplasma contamination. For experiments, unconstrained cell culture was performed within gelatin-coated 8-well µ-slides (Ibidi) and micropatterned culture was performed on 1 cm^2^ or 4 cm² (flowers, large and hollow ellipse) patterned Ibidi plastic coverslips (custom fabricated, see below) placed in the bottom of a 4-well or 6-well plates, respectively (Nunc). For both patterned and unconstrained culture, 500 µl/cm² of medium was provided to the cells.

### ESC micropatterning

Micropatterned chips were fabricated using untreated IbiTreat plastic slides (Ibidi, IB-10813) as the base substrate. Hydrophobic plastic slides were placed in contact with a 1× Master quartz anti-reflective chromium photomask (Toppan Photomask) and then exposed to deep UV light using UVO cleaner (Model No. 42-220, Jelight, USA) for 8 min at power 6 mW/cm2, λ 190 nm at a distance of 2 cm from the lamp. Slides were then incubated overnight on a drop of coating solution on Parafilm within a humidified chamber at 4°C. The coating solution consisted of 500 µg/ml of Pluronic F-127 (Sigma, P2443) and 1 mg/ml of gelatin (Sigma, G1890) dissolved in PBS. Micropatterned chips were rinsed twice with sterile PBS (Gibco) just prior to cell seeding. The seeding procedure consisted in laying down a drop of cell suspension (2.5×10^4^ cells/ml) on top of the micropatterned chip (250 µl/1 cm^2^) and leaving the cells to adhere for 1 h in the incubator. Finally, the excess of cells was removed with two successive washes using warm medium.

### Real-time PCR

RNA was extracted from ESCs using the Quick-RNA kit (Zymo Research). One microgram of RNA was reverse-transcribed using the Superscript II reverse transcriptase and oligodT_(12-18)_ (Invitrogen). qPCR was performed using a Light Cycler LC 1.5 (Roche). Amplification was carried out as recommended by the manufacturer. Twelve microlitres of reaction mixture contained 10 µl of Roche SYBR Green I mix (including Taq DNA polymerase, reaction buffer, deoxynucleoside trisphosphate mix, SYBR Green I dye and 3 mM MgCl_2_), 0.25 µM concentration of appropriate primer and 10 ng of diluted cDNA. Melting curves as well as conventional gel electrophoresis and sequencing were used to confirm the specificity of the primers. Data were analysed according to Pfaffl (2001) using ATP50 as the reference gene. Primer sequences are given in Table S1.

### Immunofluorescence

All solutions used to perform immunofluorescence in this study contained 0.01% of Pluronic F-127 (Sigma, P2443) in addition to the indicated reagents, in order to avoid dewetting of the micropatterned chips. Cells were fixed in 4% formaldehyde for 10 min at room temperature. The fixative was quenched with 50 mM ammonium chloride (Sigma) dissolved in PBS for 5 min. The cells were then incubated for a minimum of 30 min with blocking solution, which consisted of 5% donkey serum (Sigma) and 0.1% Triton X-100 (Sigma) as well as 0.03% sodium azide (Sigma). Incubation with antibodies was performed overnight at room temperature in a humidified chamber. Antibodies were all diluted in blocking solution. Primary antibodies are listed in Table S2, secondary antibodies were all Alexa Fluor conjugated (Invitrogen, A-21447, A10042, A-21202) and used at a dilution of 1/1000. Coverslips were finally mounted in ProLong Gold Antifade Mountant (Molecular Probes) 24 h prior to imaging.

### Imaging and image quantification

16-bit images were acquired using a Leica Sp8 inverted scanning confocal microscope using HyD detectors in ‘normal’ mode. We used a 40× apochromat objective with NA=1.25 and adjusted the sampling rate to obtain a voxel size of 0.38×0.38×0.5 µm. We used a scanning frequency of 700 Hz and two-frame averaging for the nuclear signal to help with subsequent segmentation. To avoid signal bleed through across channels, we used a sequential imaging strategy (405 nm and 543 nm excitation together and then 488 nm and 633 nm excitation together). The Stitching plugin (Preibisch et al., 2009) available in Fiji (Schindelin et al., 2012) was used in order to stitch multiple tiles whenever required. Images were imported inside a custom Java-based application to perform the following tasks: nuclei segmentation as well as manual correction of the segmentation, computation of nuclei 3D coordinates, computation of average intensities in colour channels and neighbours analysis. Imaging settings and image analysis parameters were set for each experiment individually and kept identical for all samples within a specific experiment. The tables of feature vectors for each individual cells within each experiment were then exported as tab-separated values for further analysis in R (R Core Team, 2013). R packages used for figure design included ggplot2 (Wickham, 2009), ggbeeswarm (https://github.com/eclarke/ggbeeswarm) and beanplot (Kampstra, 2008). R code and data tables are available in supplementary Materials and Methods. The image analysis software and segmentation tools used to detect nuclei and generate heat map figures is available on request (G.B., D. Sadurska, J. Watson, R. Portero-Migueles and S.L., unpublished).

### Calculation of the patterning score

To compute the patterning score of a colony, we computed for each cell a ‘travel distance’ from the centre of the shape, which was expressed as a percentage of the distance of the furthest shape boundary (disc radius or half-length of the ellipse main axis). The patterning score corresponds to the log_2_ of the mean travel distance of T^+^ cells over the mean travel distance of every cell. Thus, a score of 1 indicates that T^+^ cells localise on the periphery of the discs or at the tips of the ellipses. A patterning score of 0 indicates a random localisation of T^+^ cells and a negative score that T^+^ cells are closer to the centre of the shape than average.

### Quantification of surface coverage

The number of cells per cm^2^ shown in Fig. 3A was estimated at the end of the experiment by image analysis using the same images used to quantify other values such as patterning, levels of marker expression or neighbours count. For unconstrained culture, a large area (∼1 mm^2^) was imaged as described above (see also Fig. S4). The total number of segmented nuclei found within the image divided by the imaged surface area gave the global density in cm^2^. For micropatterned cultures, we first estimated the average number of cells per colony using the results of the analysis of individual colony images. Then we determined the number of shapes covered with cells on each 1 cm^2^ coverslip after formaldehyde fixation by visual inspection under a benchtop microscope (average of the result of three visual inspections). The number of cells per cm^2^ corresponded to the number of colonies on 1 cm^2^ determined by visual inspection multiplied by the average number of cells per shape.

## Supplementary Material

Supplementary information
