# Supplementary material for: Geometrical confinement controls the asymmetric patterning of brachyury in cultures of pluripotent cells
Source: Development. 2018 Sep 21;145(18):dev166025. doi: 10.1242/dev.166025 (PMC6176930; doi:10.1242/dev.166025)
Supplement: Supplementary information [file develop-145-166025-s1.pdf]

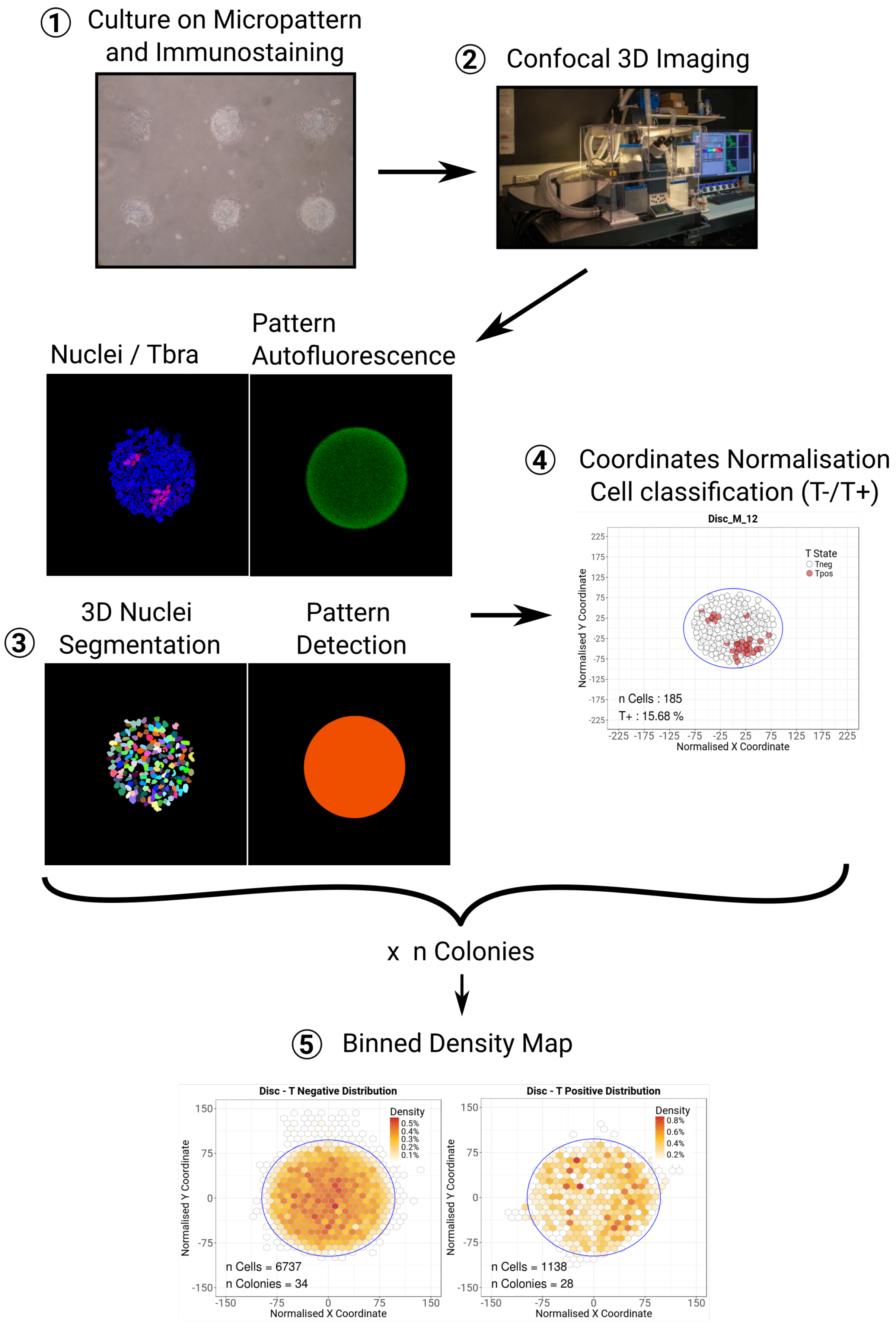


Development • Supplementary information

**Fig. S1 quantitative immunofluorescence and generation of the binned density spatial maps.**

Micropatterning technique allows to force the cells to form colonies of a defined shape and size. This enables the possibility to determine the preferential distribution of a specific subpopulation within a given geometry using the following procedure:

The cells are fixed and stained for both a marker of interest (here T is represented) and a nuclear marker (here Lamin B1) which is required to identify each individual cell (1). Imaging is performed using confocal microscopy* (2). A custom 3D nuclei segmentation method (manuscript in preparation and software available on request) allows for the automated identification of individual cells within each image (3). Once nuclei are segmented, the coordinates of the nucleus barycentre and the mean intensities of the signal in other image channels is computed. Also, the autofluorescence of the patterned substrate is segmented (3) in order to obtain the centre of the shape which is then used as a reference to normalise coordinates of the cells across multiple colonies. The cells are then classified using a threshold set manually based on the mean fluorescence intensity detected within the nucleus** (4). The binned density maps (BDM) are generated with ggplot2 using coordinates of cells accumulated over all the colonies imaged over all independent experiments (5). NB: The 'density' scale bar shown in the BDMs represent the frequency density of the 2D histogram, in other words, this represents the number of events found within a specific bin divided by the total number of event in the data.

* Due to the time required to image colonies at a resolution sufficient for accurate cell segmentation (~ 3 min / colony for ellipse M, 15 min for flowers), some form of colony sampling is required. To account for variations in background and possible staining inhomogeneities, we sampled colonies at various locations on the coverslip based on the nuclear signal (without looking at the T signal to prevent selection bias) and based on whether the colony morphology nicely followed the pattern shape visible by autofluorescence (the staining procedure sometimes led to colonies detaching from the pattern or to colonies with a sheared structure). The number of colonies imaged per condition largely depended on the number of undamaged colonies as well as time considerations.

** Selecting a threshold to define cells as positive or negative for a specific marker always contains a part of subjectivity (even when using a statistical method such as fitting a gaussian mixture as the choice of the method also requires a number of assumptions).

To select thresholds as objectively as possible, we created a scatter plot of the distribution of intensities for all the cells in the experiment independently of the culture condition. Our software allows for clicking on individual data points to visualise a thumbnail image of the corresponding cell. This permitted to iteratively refine the threshold based on both the shape of the distribution of intensities as well as based on the image visualisation while remaining blind to the sample under scrutiny for each click.

Development • Supplementary information

| Shape | Pitch | Area of 1 pattern (µm²) | Shape number (1 cm²) | Adhesive Area (µm²) | Adhesive Area (% Unpatterned) |
|---|---|---|---|---|---|
| Disc M | 400 | 30000 | 256 | 7680000 | 7,68 % |
| Ellipse M | 400 | 30000 | 240 | 7200000 | 7,20 % |
| Ellipse L | 400 | 90000 | 153 | 13770000 | 13,77 % |
| Hollow Ellipse | 400 | 90000 | 128 | 11520000 | 11,52 % |
| Flower | 600 | 360000 | 16 | 5760000 | 5,76 % |

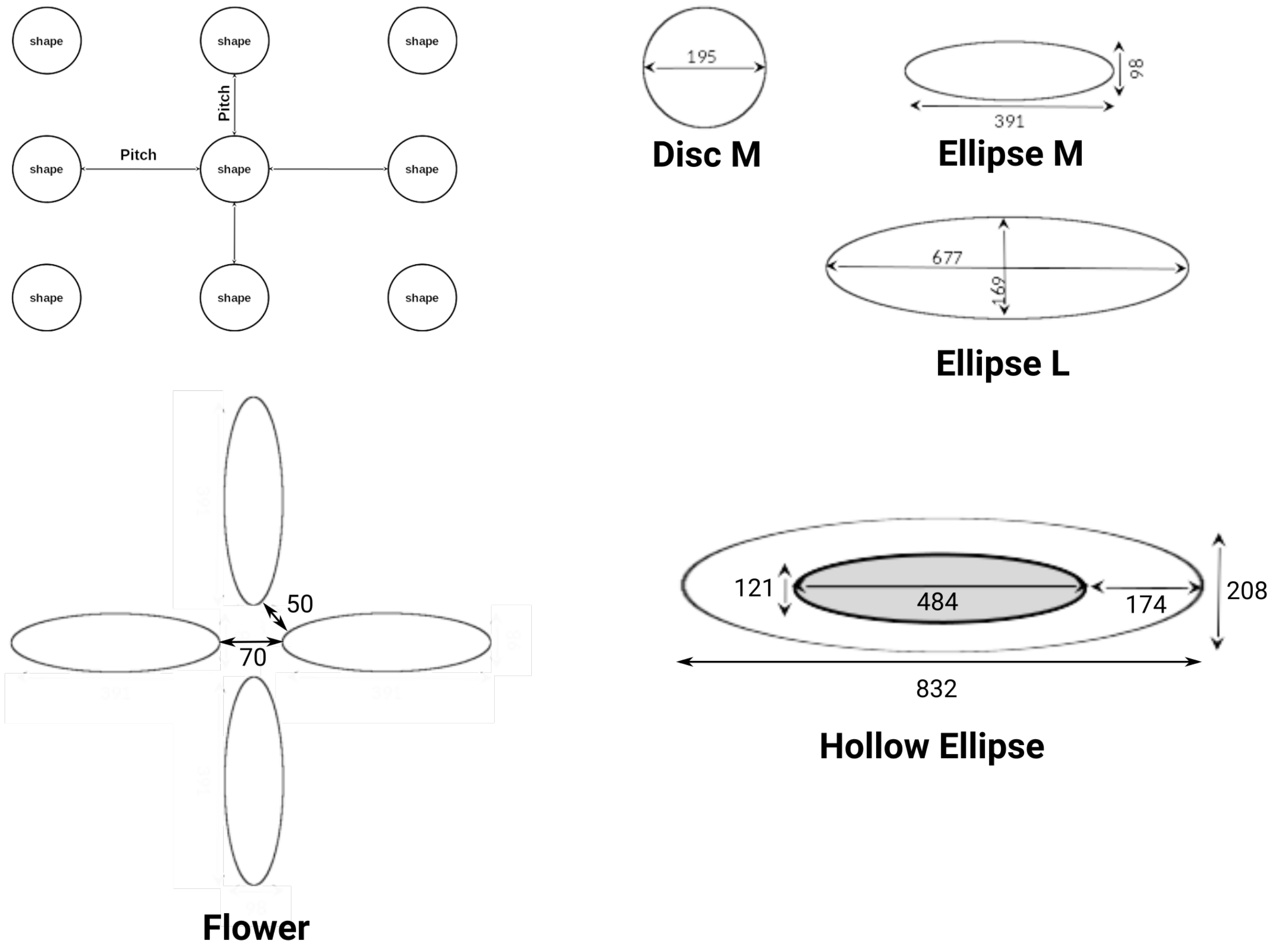


**Fig. S2 Dimensions and theoretical surface coverages of pattern shapes used in this study.**

The upper table summarises the properties of each geometrical design used in this study, including the edge to edge distance between shapes in both the x and y axis regardless of the geometry (pitch), the surface area of one shape, the total adhesive surface for one slide, the number of shapes on 1cm$^2$ chip and the percentage that this area represents compared to the area of a fully adhesive chip. Below the table, shapes are drawn and dimensions are indicated in µm.

Development • Supplementary information

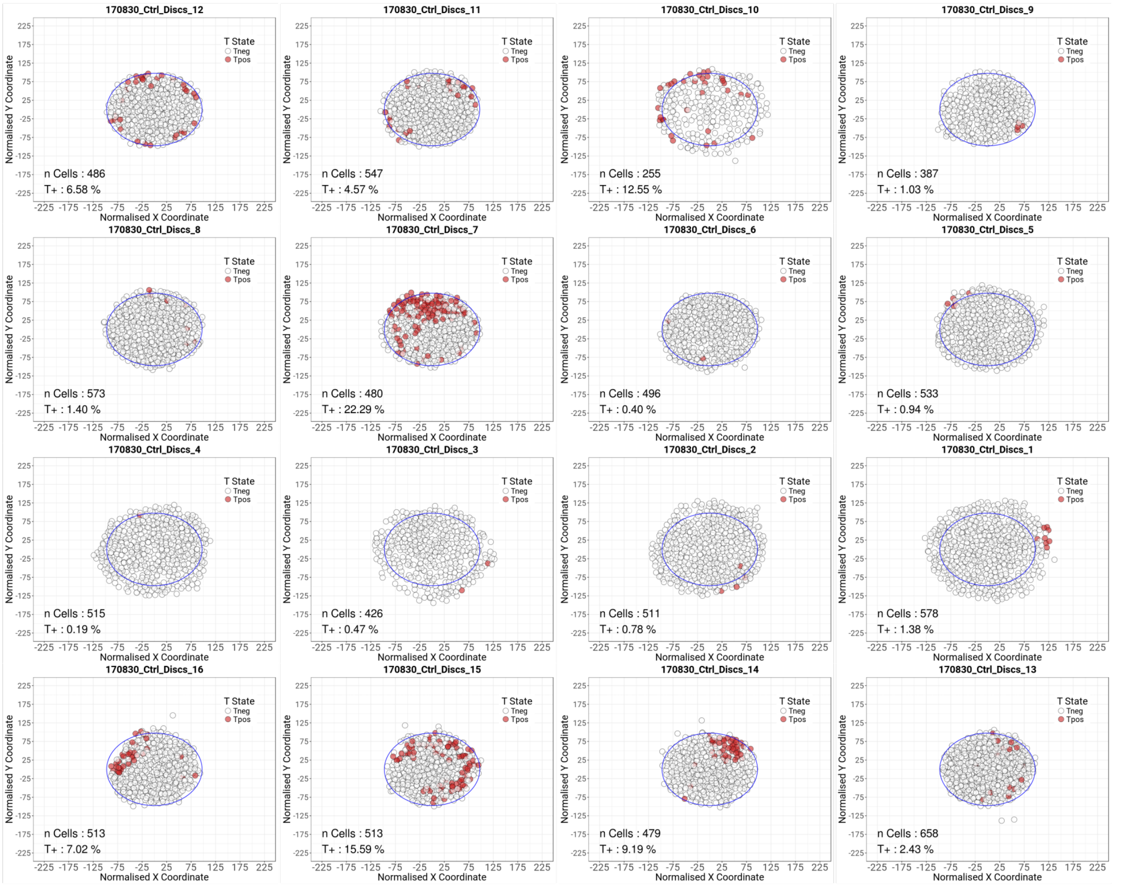
170830_Ctrl_Discs_12
n Cells : 486
T+ : 6.58 %
170830_Ctrl_Discs_11
n Cells : 547
T+ : 4.57 %
170830_Ctrl_Discs_10
n Cells : 255
T+ : 12.55 %
170830_Ctrl_Discs_9
n Cells : 387
T+ : 1.03 %
170830_Ctrl_Discs_8
n Cells : 573
T+ : 1.40 %
170830_Ctrl_Discs_7
n Cells : 480
T+ : 22.29 %
170830_Ctrl_Discs_6
n Cells : 496
T+ : 0.40 %
170830_Ctrl_Discs_5
n Cells : 533
T+ : 0.94 %
170830_Ctrl_Discs_4
n Cells : 515
T+ : 0.19 %
170830_Ctrl_Discs_3
n Cells : 426
T+ : 0.47 %
170830_Ctrl_Discs_2
n Cells : 511
T+ : 0.78 %
170830_Ctrl_Discs_1
n Cells : 578
T+ : 1.38 %
170830_Ctrl_Discs_16
n Cells : 513
T+ : 7.02 %
170830_Ctrl_Discs_15
n Cells : 513
T+ : 15.59 %
170830_Ctrl_Discs_14
n Cells : 479
T+ : 9.19 %
170830_Ctrl_Discs_13
n Cells : 658
T+ : 2.43 %
T State
Tneg
Tpos
Normalised X Coordinate
Normalised Y Coordinate

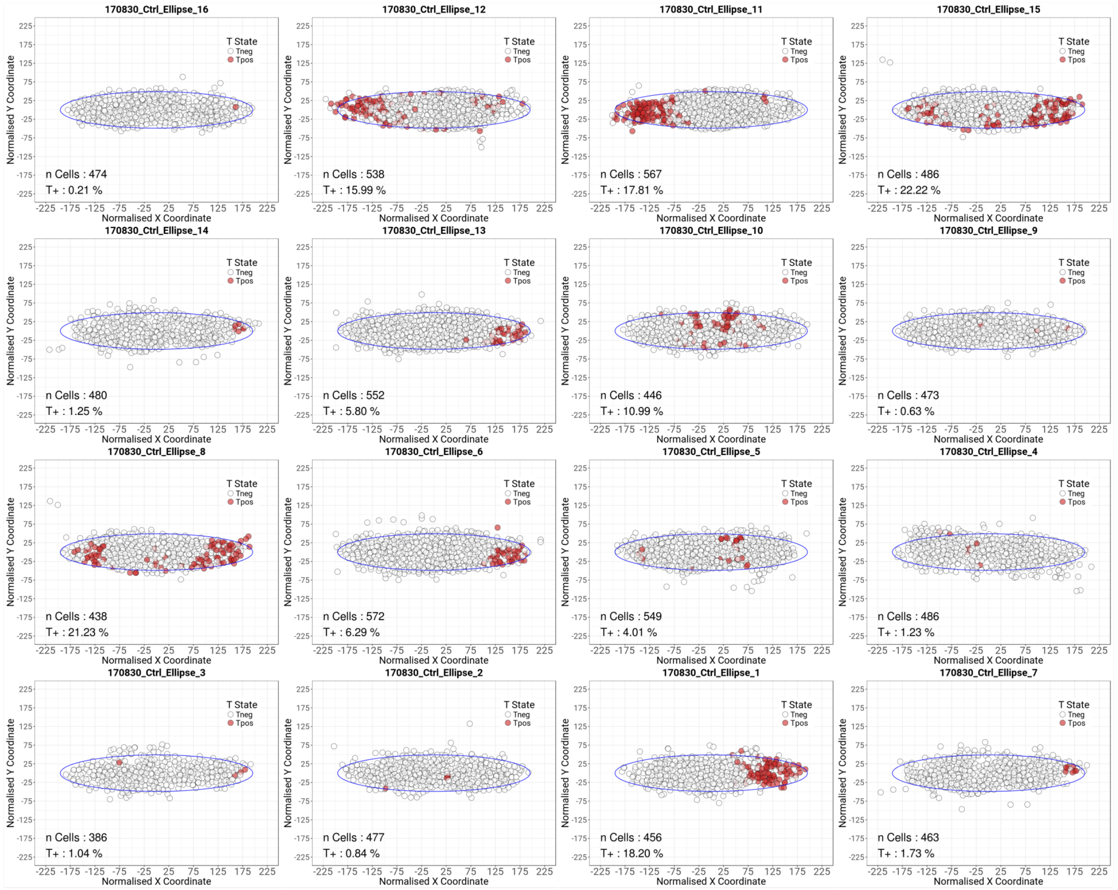
170830_Ctrl_Ellipse_16
n Cells : 474
T+ : 0.21 %
170830_Ctrl_Ellipse_12
n Cells : 538
T+ : 15.99 %
170830_Ctrl_Ellipse_11
n Cells : 567
T+ : 17.81 %
170830_Ctrl_Ellipse_15
n Cells : 486
T+ : 22.22 %
170830_Ctrl_Ellipse_14
n Cells : 480
T+ : 1.25 %
170830_Ctrl_Ellipse_13
n Cells : 552
T+ : 5.80 %
170830_Ctrl_Ellipse_10
n Cells : 446
T+ : 10.99 %
170830_Ctrl_Ellipse_9
n Cells : 473
T+ : 0.63 %
170830_Ctrl_Ellipse_8
n Cells : 438
T+ : 21.23 %
170830_Ctrl_Ellipse_6
n Cells : 572
T+ : 6.29 %
170830_Ctrl_Ellipse_5
n Cells : 549
T+ : 4.01 %
170830_Ctrl_Ellipse_4
n Cells : 486
T+ : 1.23 %
170830_Ctrl_Ellipse_3
n Cells : 386
T+ : 1.04 %
170830_Ctrl_Ellipse_2
n Cells : 477
T+ : 0.84 %
170830_Ctrl_Ellipse_1
n Cells : 456
T+ : 18.20 %
170830_Ctrl_Ellipse_7
n Cells : 463
T+ : 1.73 %
T State
Tneg
Tpos
Normalised X Coordinate
Normalised Y Coordinate

**Fig. S3 Positioning of T+ cells within individual ESC colonies grown on micropatterns.**

Examples of cells distributions within individual ESC colonies grown on discs or ellipses micropatterns. 3D coordinates of the cells barycenters are projected on the XY plane and represented as circles. The blue line indicates the boundary of the micropattern. The percentage of T+ cells and the total number of cells (n Cells) is indicated for each plot. NB: All individual colony plots may be generated using the provided data text files and R code.

Development • Supplementary information

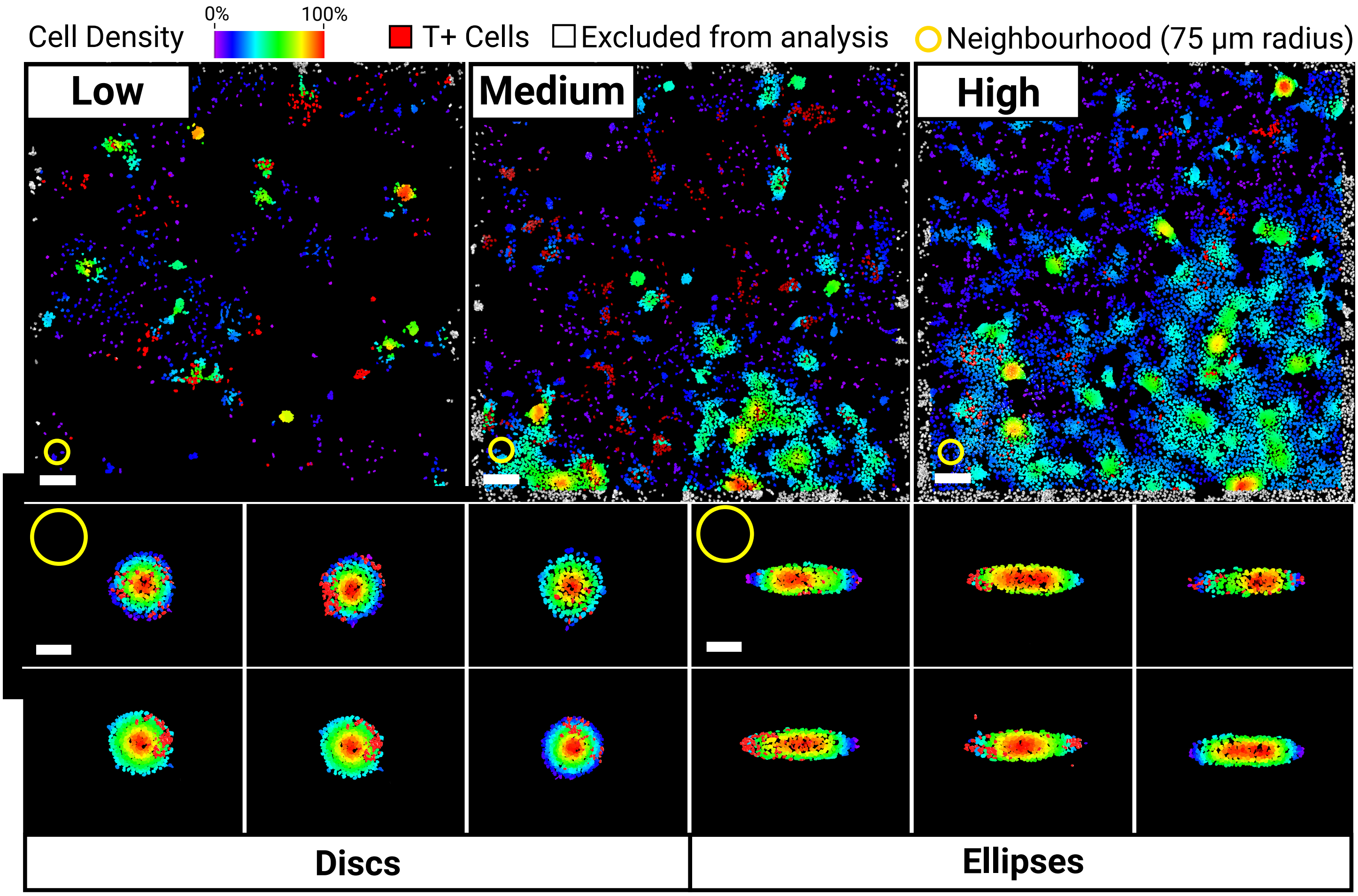


**Fig. S4 Local cell density distributions**

Representative heatmaps of the distribution of local cell density computed in a circular region of 75 µm of radius around each cell for ESC cultured at low, medium and high density or grown on disc or ellipse micropatterns. Densities range from 0 to 100 % of the maximum neighbour count identified in the image. T+ cells are shown as bright red cells and cells that are excluded from the neighbourhood analysis due to their proximity with the image border are shown in white. Scale bar: 200µm for low, medium and high densities, 100µm for Disc and Ellipse micropatterns.

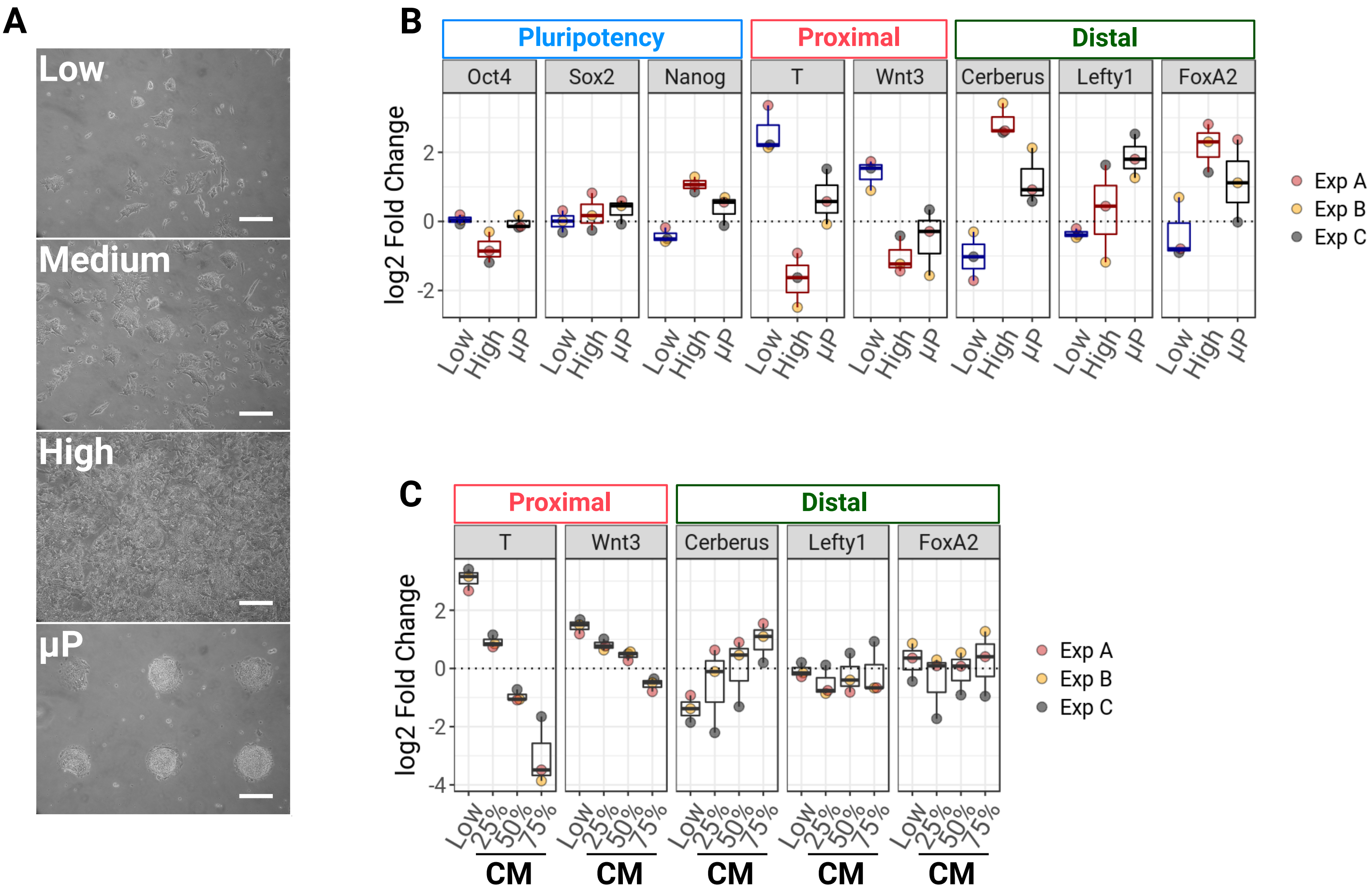


**Fig. S5 Bulk cell density modulates the balance between posterior and anterior identities.**

**A** sample wide field micrographs of ESC cultured at low, medium and high densities or on disc M micropatterns, scale bar: 200µm. **B, C** qPCR results are represented as box plots generated from 1 technical replicate for each of 3 independent experiments (indicated as colour coded dots). The y axis indicates the log2 of the fold change in transcript levels when compared to the reference sample (set to 0 for each individual experiment - dotted line). The reference sample consisted in cells cultured at medium density. **B** compares the levels of pluripotency, proximal and distal genes for cells cultured at low, medium, high density or on discs micropatterns. **C** shows the evolution of transcripts levels for cells cultured at low density with increasing amounts of medium conditioned for 48h at high density (percentage on the x axis).

Development • Supplementary information

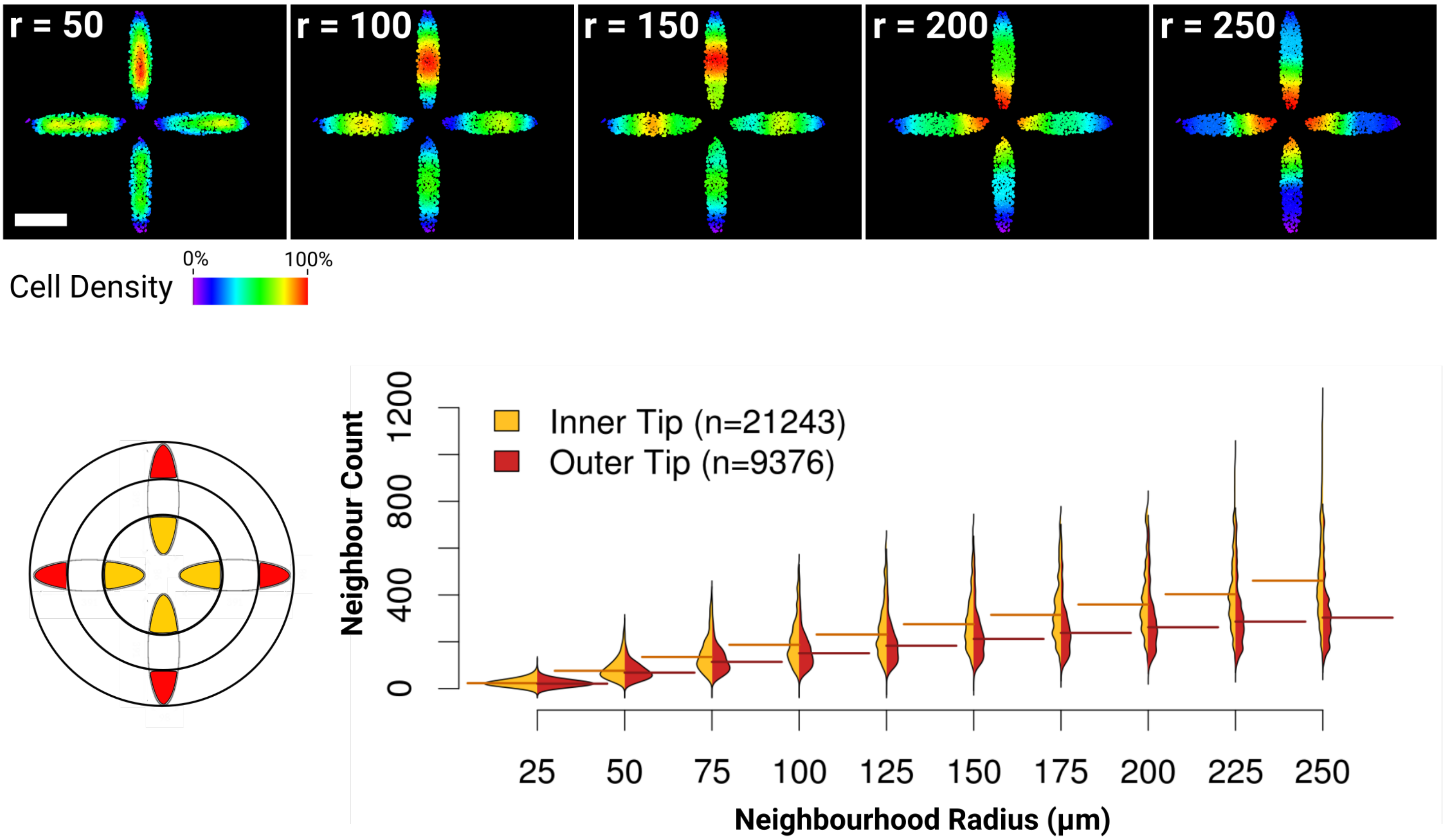


**Fig. S6 Colonies in close proximity neither influence the variability in T expression nor T patterning**

Top panel: Heatmaps of a representative flower pattern. Each image represents the distribution of local cell densities computed using circular regions of increasing radius (shown in the upper left corner, r: neighborhood radius in µm), scale bar: 200µm.

Bottom panel: Split bean plots showing the distribution of the neighbour count for the inner tip cells (orange) and outer tip cells (red) computed using circular regions of increasing radii. Results include 3 independent experiments. The tips here were defined by splitting each ellipses into 3 sections of equal main axis length (left diagram). Notice that the difference between inner tips versus outer tips becomes apparent from around 100µm therefore this design tests for effects of morphogen gradients with a diffusion range of 100µm or above.

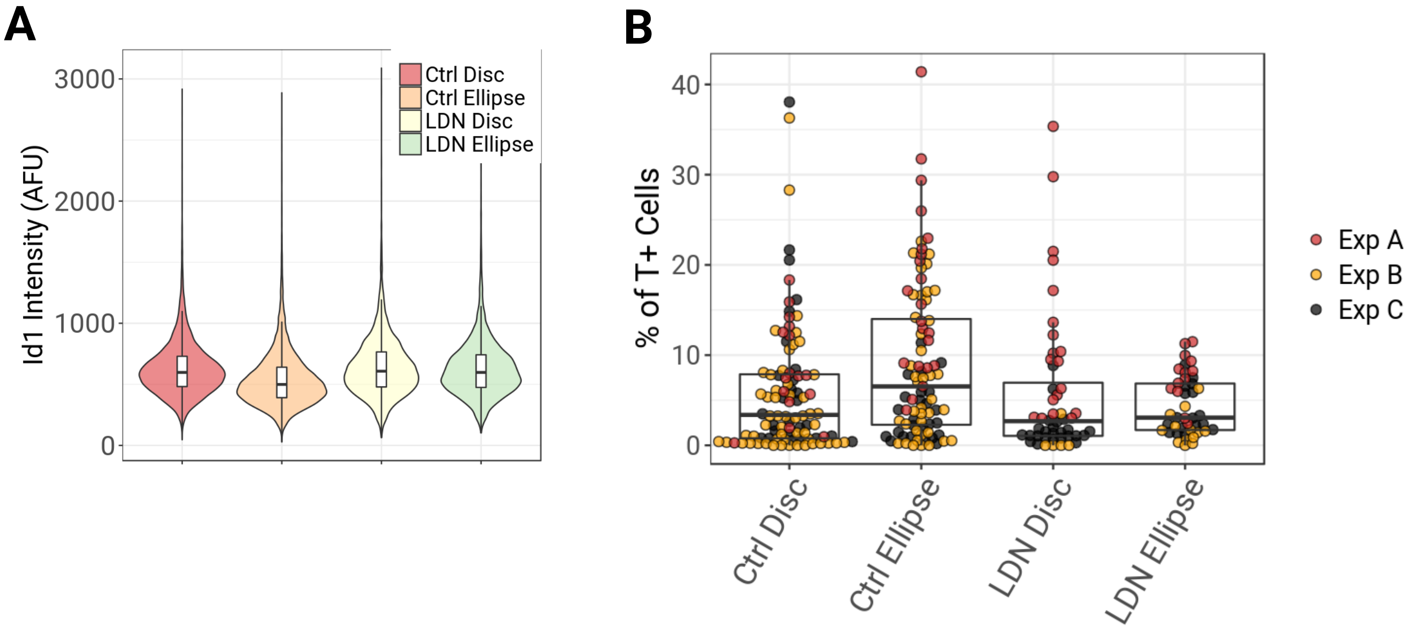


**Fig. S7 BMP signalling is not required for T expression**

Distributions of the mean intensity levels of Id1 detected by qIF **(A)** and of the percentages of T+ cells found within individual colonies **(B)** for cells grown on micropatterns with or without 48h LDN treatment (100nM).

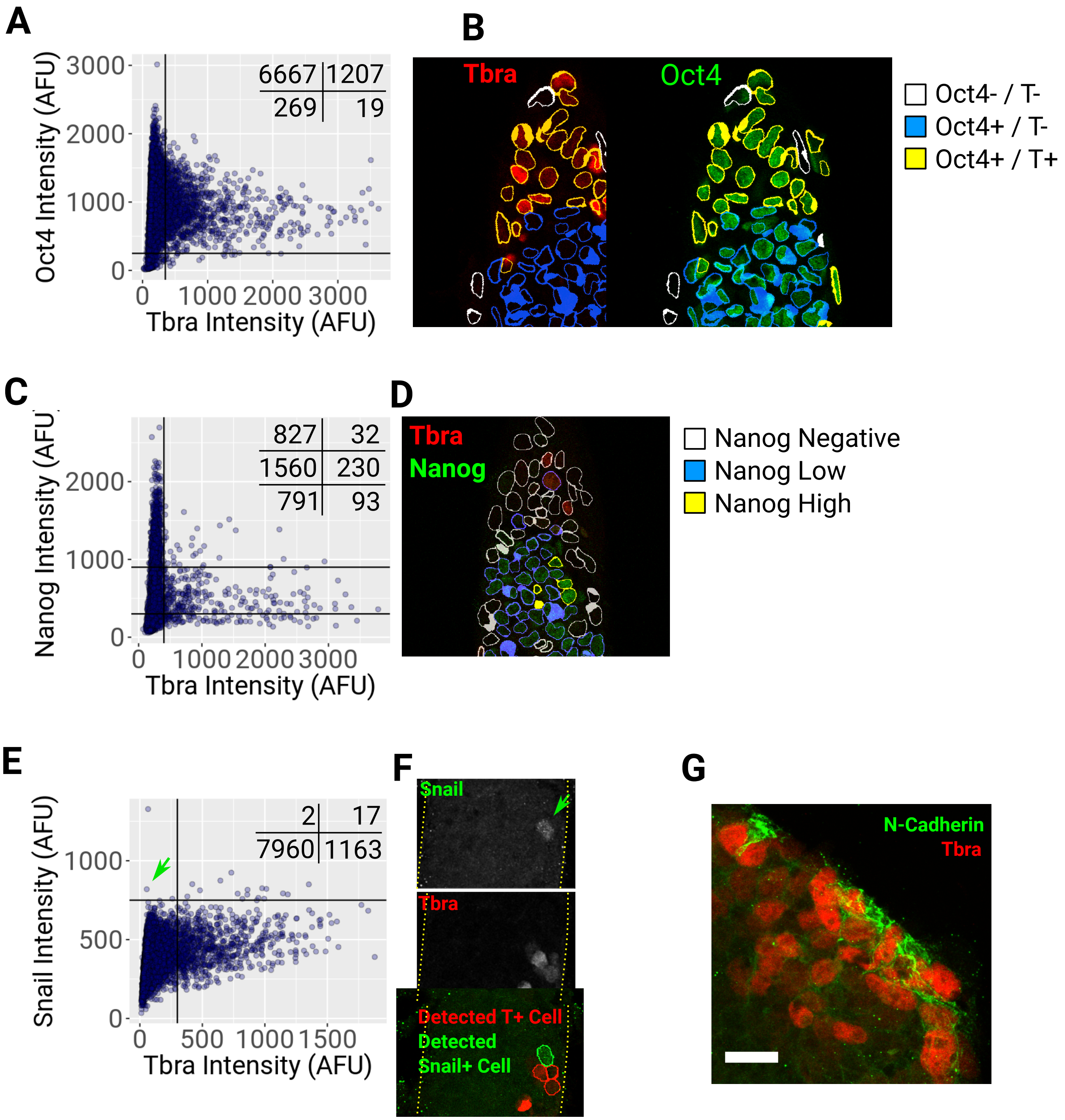


Development • Supplementary information

**Fig. S8 T+ cells represent a pre-ingressing population**

**A, C, E** Representative scatter plots of intensities of T and a marker of interest as indicated for cells grown on ellipse micropatterns. **B, D** Confocal z section images of a T (red) and Oct4 (B-green) or Nanog (D-green) co-staining. The outline of detected cells is represented and colour coded according to the Oct4 or Nanog phenotype. **F** Confocal z section image of a T and Snail co-staining.. Individual channels are shown as grayscale image to emphasize that T+ cells are Snail- and that Snail+ cells are very rare. The green arrow shows one Snail+ cell on the Snail channel image and the corresponding data point on the scatter plot. **G**
Max projection of a representative confocal image of a T (red) and N-Cadherin (green) co-staining. Please note that quantification of the number of N-Cadherin positive cells was not possible due to the membranar localisation of this marker and therefore only characteristic examples of rare incidences of N-Cadherin staining are shown. Scale bar: 20 µm.

**Supplementary Material : R source code and data**

This .zip archive contains the source code and the data tables required to reproduce the charts included in this article. Instructions for use are included in the 'Readme.md' file contained in the archive.

Click here to Download the .zip file

**Table S1. Real-time PCR primers list**

| Gene | Primer sequence | |
|---|---|---|
| ATP50 | F | CTATGCAACCGGCCTGTACT |
| | R | GATGATACGGTGGGTGTTGC |
| Nanog | F | AAGTTTTGCTGCAACTGTACG |
| | R | CCCCAGGGCTATCTGGTGAAC |
| Oct4 | F | TCAGCTTGGG CTAGAGAAGG |
| | R | TGACGGGAACAGAGGGAAAG |
| Sox2 | R | CTCCGGAAGCGTGTACTTA |
| | F | CACAACTCGGAGATCAGCAA |
| Brachyury | F | GACTTCGTGACGGCTGACAA |
| | R | CGAGTCTGGGTGGATGTAG |
| Wnt3 | F | GATGCCCGCTCAGCTATGAA |
| | R | CGGAGGCACTGTCGTACTTG |
| Lefty1 | F | CCAACCGCACTGCCCTTAT |
| | R | CGCGAAACGAACCAACTTGT |
| Cerberus | R | CTCTGGGGAAGGCAGACCTAT |
| | F | CCACAAACAGATCCGGCTT |
| FoxA2 | F | CCCTACGCCAACATGAACTCG |
| | R | GTTCTGCCGGTAGAAAGGGA |

**Table S2. List of primary antibodies used in this study**

| Gene | Host | Dilution | Reference |
|---|---|---|---|
| LaminB1 | Rabbit | 1/1000 | ab16048 |
| Oct4 | Mouse | 1/400 | sc-5279 |
| Id1 | Rabbit | 1/200 | Biocheck 37-2 |
| Tbra | Goat | 1/400 | R&D AF2085 |
| Snail1 | Mouse | 1/50 | Gift from Herreros Lab (Francí et al., 2006) |
| Nuclear Pore Complex | Mouse | 1/1000 | Abcam, ab24609 |
| Nanog | Rat | 1/200 | eBioMLC-51 |
| Active β-Catenin | Mouse | 1/1000 | Millipore 05-665 |
| N-Cadherin | Mouse | 1/400 | Sigma C3865 |
